# Pearl powder reduces sleep disturbance stress response through regulating proteomics in a rat model of sleep deprivation

**DOI:** 10.1111/jcmm.15095

**Published:** 2020-03-27

**Authors:** Meng Xia, Delun Huang, Yuangming Tong, Jiang Lin

**Affiliations:** ^1^ School of Basic Medicine Guangxi University of Chinese Medicine Nanning China; ^2^ Information Center Guangxi Institute of Chinese Medicine and Pharmaceutical Science Nanning China

**Keywords:** cognition, hippocampus, pearl, proteomics, sleep deprivation

## Abstract

**Aims:**

This study aimed to explore whether pearl could help prevent cognitional morbidity and improve the metabolic processes of hippocampus.

**Methods:**

Rats were divided into group of control (CTL), sleep deprivation (SD) and pearl powder (PP). The sleeplessness was introduced to all rats except control. Before and after administration with vehicle or pearl powder, cognition was evaluated by Morris water maze (MWM). The protein expression in hippocampus among all groups was examined using iTRAQ‐based global proteomic analysis.

**Results:**

Morris water maze tests revealed improvements of insomnia‐induced cognitive deficit in both PP‐ and ES‐treated rats, as compared to SD rats. However, proteomic analysis indicates that the pharmacological impact on gene expression of these two medicines is quite different: pearl is more capable of correcting aberrant gene expression caused by SD than estazolam. Therefore, pearl is more suitable for treatment of insomnia. These data, together with protein‐protein interaction analysis, indicate that several pathways, affected by sleep deprivation, may be rescued by pearl powder: retrograde endocannabinoid signalling pathway, and the protein interaction or network enrich in oxidative phosphorylation Parkinson's disease and Huntington disease, etc

**Conclusions:**

Sleep deprivation can mimic cognition decline caused by insomnia with altered protein expression in the hippocampus; such behavioural and pathological changes can be significantly ameliorated by pearl powder.

## 1 INTRODUCTION

Pearl, in addition to improving skin beauty, is a well‐known traditional Chinese medicine (TCM) for various disorders, including insomnia, palpitations, convulsions or epilepsy. According to Compendium of Materia Medica, a famous historical Chinese Herb Pharmacology, patients in stressful status can take pearl powder as tranquillizer and sedative to protect and nourish YIN by calming endogenous WIND and removing toxic substances.^1^, ^2^ Pearl is mainly composed of calcium carbonate and magnesium carbonate, which accounted for 91% of total weight. Other inorganic molecules such as silica, calcium phosphate, aluminium oxide and ferric oxide, as well as some trace elements such as sodium, magnesium, manganese, selenium, aluminium and copper can also be found in pearl powder. It also contains essential amino acids including lysine (Lys), valine (Val), threonine (Thr), methionine (Met), leucine (Leu), phenylalanine (Phe), tryptophan (Trp) and histidine (His).^3^, ^4^ The nutrients in pearl powder are enriched with proteins, peptides and amino acids, making great contributions to the body's bioactivity including improving antioxidant defence system.^5^ For example, amino acid Asp and Glu possess antioxidant properties, and Cys exhibits free radical quenching ability.^6^ Indeed, there are different usages of pearl when it is added to different combination of Chinese herbs which can selectively targeting different part of body sites to balance YIN.^7^

Estazolam, a triazolobenzodiazepine, has been widely used as hypnotic medication.^8^, ^9^ It belongs to class of benzodiazepines, a GABAa receptor agonist in the brain. Estazolam can cause sedation and relaxation, therefore facilitating sleep. However, estazolam is extremely addictive with very severe withdrawal symptoms and can cause a similar black‐out effect, and some studies reported that it also confers the risk of dementia.^10^, ^11^ Therefore, pearl and nacre are valuable traditional medicines with a lot of potential for more clinical treatments, if we understand their mechanism of action and signalling pathway.

In this study, we have been suggested that pearl contains many bio‐protective reagents that target several molecules and play critical roles in multiple metabolic pathways. To test this hypothesis, we used a previously validated insomnia model in rats, induced by sleep deprivation (SD), resulting in learning and memory impairment which had been thought to be caused by pathological changes in hippocampus.^12^, ^13^ Based on it, we conducted an advance technology iTRAQ for a broad screening and comparing on differential proteomic expression in hippocampus. Our results indicate that sleep deprivation can induce learning and memory deficit which associated with oxidative phosphorylation, ribosome and proteasome proteins. Pearl is able to lowering or reversing some, if not all of the mal‐alternation.

## 2 METHODS

### 2.1 Animals and ethics statement

The experimental protocols, care and handling of animals used in this study were approved by the Institutional Animal Care and Use Committee at Guangxi University of Chinese Medicine, in accordance with IASP Guidelines for the Use of Animals in Research. Male Sprague Dawley (SD) rats were purchased from Hunan Slack Jingda Experimental Animal Co. Ltd, Hunan, China, with the average body weight of 180 ± 20 g. Rats were housed in four rats per cage in a well‐ventilated colony room having a 12‐hour (h) light/dark cycle (lights on at 7:00 am) and temperature of 22°C.

**Figure 1:**
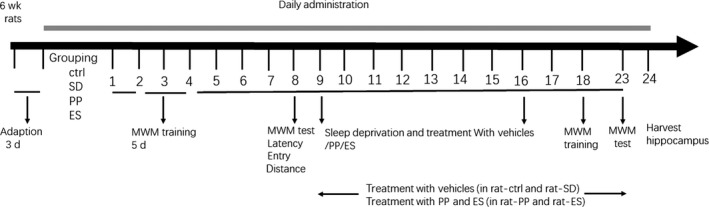
Workflow. Six‐week‐old rats were conducted MWM test from day 3 to day 7 with the hidden platform in the water tank, and then, spatial memory and cognition were examined by MWM at day 8 without the hidden platform. From day 9 to day 16, all rats were administrated with or without sleep deprivation, as well as treatment, respectively, with PP/ES or saline from day 9 to day 23. Pearl powder (2.5 mg/kg/d) and estazolam (0.13 mg/kg/d) in saline, the same volume of saline was administered in groups of CTL and SD. Afterwards, the MWM training (days 18‐22) and test (day 23) were performed for another 6 d again. Then, the rats were killed and hippocampus tissues were collected for proteomic and other analyses

Six‐week‐old rats received standard laboratory rat chow and tap water ad libitum feeding. After a week of adaptation, rats were randomly assigned into four groups (n = 9/group) as control (CTL), sleep deprivation (SD), pearl powder (PP) and estazolam (ES). The sleep deprivation was conducted for about 20 hours/d (23:00‐19:00) and lasted for total 7 days: rats were placed in a platform (height 20 cm and 5 × 5 square centimetre area) which was in a container of water (depth 18 cm). The Ctr, PP and ES groups were administrated repeatedly with 0.9% saline, pearl powder (2.5 mg/kg/d) and estazolam (0.13 mg/kg/d) starting from 1st day of sleep deprivation once per day for 14 days. The schedule of administration is illustrated in Figure 1.

### 2.2 Morris Water Maze (MWM) test

Before behavioural tests, rats were adapted in our animal facility for at least 1 week in the housing room which has the same condition as behavioural room.^14^ MWM training and test were performed in a circular water tank (120 cm in diameter) containing opaque water (22 ± 1°C) at a depth of 25 cm and were divided into four quadrants. A hidden escape platform (9 cm in diameter) was placed in the centre of one quadrant, with its surface 1 cm below the water. The rats were subjected to an acquisition trial four times a day for five consecutive days. During each trial, the rats were placed in water at one of the four quadrants and the starting position was randomly selected. Each rat was trained to swim for locating the hidden platform. Rats that failed to find the hidden platform within 60 seconds were placed on it for 30 seconds. The same platform location was used for all rats. The platform was removed on the sixth day, and the rats were subjected to the spatial probe trial test for 60 seconds. The time and distance spent in the target quadrant were recorded.

### 2.3 Protein extraction and trypsin digestion

Rats were killed in a box connected to CO^2^ tank. Then, rat brains were quickly removed from skulls and left on cold platform. Two pieces of hippocampal tissues underneath cortex were carefully dissected out using surgery tools, immediately frozen on dry ice and stored in −80°C freezer. Sample size: 3 rats per group. The hippocampus tissues were homogenized thoroughly with a tissue grinder in 1:50 (W/V) Lysis Buffer (8 mol/L urea, 2 mmol/L EDTA, 10 mmol/L DTT and 1% Protease Inhibitor Cocktail). Samples were sonicated for 3 minutes and centrifuged at 13 000 *g* at 4℃ for 10 minutes to remove debris, and the protein supernatant was collected and precipitated with 3× Volume of ice‐cold acetone for 3 hours at −20℃. After centrifugation at 4°C at 12 000 *g* for 10 minutes, the protein in the pellet was resuspended with urea buffer (8 mol/L urea, 100 mmol/L TEAB). The protein concentration was determined using a Modified Bradford Protein Assay Kit according to the manufacturer's instructions. After that, 100 μg protein of each sample was first reduced with 10 mmol/L DTT at 37°C for 60 minutes and then alkylated with 55 mmol/L iodoacetamide (IAM) at room temperature for 30 minutes in the dark. The urea concentration of protein sample was diluted to less than 2 mol/L by adding 100 mmol/L TEAB. The total protein of each sample was digested with Sequencing Grade Modified Trypsin at the mass ratio of protein:trypsin = 50:1 at 37°C overnight and at a ratio of 100:1 for a second digestion at 4 hours.

### 2.4 Peptide isobaric labelling and HPLC fractionation

After trypsin digestion, peptide was desalted by Strata‐X SPE column and vacuum‐dried. Peptide was reconstituted in 20 μL 500 mmol/L TEAB and processed according to the manufacturer's protocol for 8‐plex iTRAQ kit. Briefly, one unit of iTRAQ reagent was all added to peptide solution after thawed and dissolved in 50 μL isopropanol. The peptide mixtures were incubated for 2 hours at room temperature, and then pooled and dried by vacuum centrifugation. The dried and labelled peptide was reconstituted with HPLC solution A (2% ACN, pH 10) and then fractionated into fractions by high pH reverse‐phase HPLC using Waters Bridge Peptide BEH C18 (130 Å, 3.5 μm, 4.6 * 250 mm). Briefly, peptides were first separated with a gradient of 2%‐98% acetonitrile in pH 10 at a speed of 0.5 mL/min over 88 minutes into 48 fractions. Then, the peptides were combined into 12 fractions and dried by vacuum centrifugation. The peptide fractions were desalted using ZipTip C18 according to the manufacturer's instructions. Samples were finally dried under vacuum and kept at −20°C until MS analyses were performed.

### 2.5 High‐resolution LC‐MS/MS analysis

This experiment was performed by NanoLC 1000 LC‐MS/MS using a Proxeon EASY‐nLC 1000 coupled to Thermo Fisher Q Exactive. Trypsin digestion fractions were reconstituted in 0.1% FA and directly loaded onto delivered to a reversed‐phase pre‐column (Acclaim PepMap®100 C18, 3 μm, 100 Å, 75 μm × 2 cm) at 5 μL/min in 100% solvent A (0.1 mol/L acetic acid in water). Next, peptides eluted from the trap column were loaded onto a reversed‐phase analytical column (Acclaim PepMap® RSLC C18, 2 μm, 100 Å, 50 μm × 15 cm). The gradient was comprised of an increase from 15% to 35% solvent B (0.1% FA in 98% ACN) over 45 minutes, 35% to 98% solvent B during 5 minutes and keep in 98% in 5 minutes at a constant flow rate of 300 nL/min on an EASY‐nLC 1000 system. The eluent was sprayed via NSI source at the 2.0 kV electrospray voltage and then analysed by tandem mass spectrometry (MS/MS) in Q Exactive. The mass spectrometer was operated in data‐dependent mode, automatically switching between MS and MS/MS. Full‐scan MS spectra (from m/z 350 to 1800) were acquired in the Orbitrap with a resolution of 70 000. Ion fragments were detected in the Orbitrap at a resolution of 17 500, and the 20 most intense precursors were selected for subsequent decision tree‐based ion trap HCD fragmentation at the collision energy of 27% in the MS survey scan with 45.0‐second dynamic exclusion.

### 2.6 Proteomic data analysis and bioinformatics

The resulting MS/MS raw data were searched against the Rattus norvegicus proteome database (taxon identifier: 10 116 including 29 975 protein sequences) downloaded from UniProt database using Sequest software integration in Proteome Discoverer (version 1.3, Thermo Scientific). Trypsin was chosen as enzyme, and two missed cleavages were allowed. Carbamidomethylation (C) was set as a fixed modification and oxidation (M), and acetylation in N‐term was set as variable modification. The searches were performed using a peptide mass tolerance of 20 ppm and a product ion tolerance of 0.05 D, resulting in 1% false discovery rate (FDR). The GO (gene ontology) was used for the annotation of the identified proteins, which was composed of BP (biological processes), CC (cellular components) and MF (molecular functions), and the analysis was based on the UniProt‐GOA database (http://www.ebi.ac.uk/GOA/). The pathway analysis of differently expressed proteins was based on the KEGG (Encyclopedia of Genes and Genomes) database. The online KEGG Automatic Annotation Server (KAAS) service tools were used to annotate the KEGG database description for each protein and to map the annotation results using other KEGG online service tools and the KEGG mapper. The interaction between differential expression protein (DEPs) groups was derived from the Search Tools for the Retrieval of Interacting Genes/Proteins (STRING) database. A high confidence (0.7) for the required interaction score and the active interaction sources, including text mining, experiments and databases, were chosen to draw the protein‐protein interaction map using Cytoscape 3.2.1.

### 2.7 Western blotting

The protein expression levels of PLK1, PGP and HGS were analysed by a Western blot. Equal amount of protein from each sample was loaded onto SDS‐PAGE (sodium dodecyl sulphate polyacrylamide gel electrophoresis) gel, separated by electrophoresis and transferred onto a polyvinylidene difluoride (PVDF) membrane by a semidry Western blot system (Trans‐Blot® TurboTM System, Bio‐Rad, Singapore). The membrane was then blocked in 5% non‐fat milk for 2 hours at 37°C and incubated at 4 ℃ with primary antibodies for 16‐18 hours Membranes were washed three times using TBST buffer (a mixture reagent of TBS [Tris‐buffered saline] and Tween‐20) and then incubated with secondary antibodies in TBST buffer for 2 hours at 37°C. Bands were visualized with an alkaline phosphatase detection kit (C3206, Beyotime Biotechnology Inc). The following commercially available antibodies were used as primary antibodies: (anti‐RIMS3 antibody, anti‐CPI‐17, anti‐metabotropic glutamate receptor 2 antibody, anti‐superoxide dismutase 1 antibody, anti‐PP2A beta antibody, anti‐beta‐actin antibody).

### 2.8 Statistical analysis

**Figure 2:**
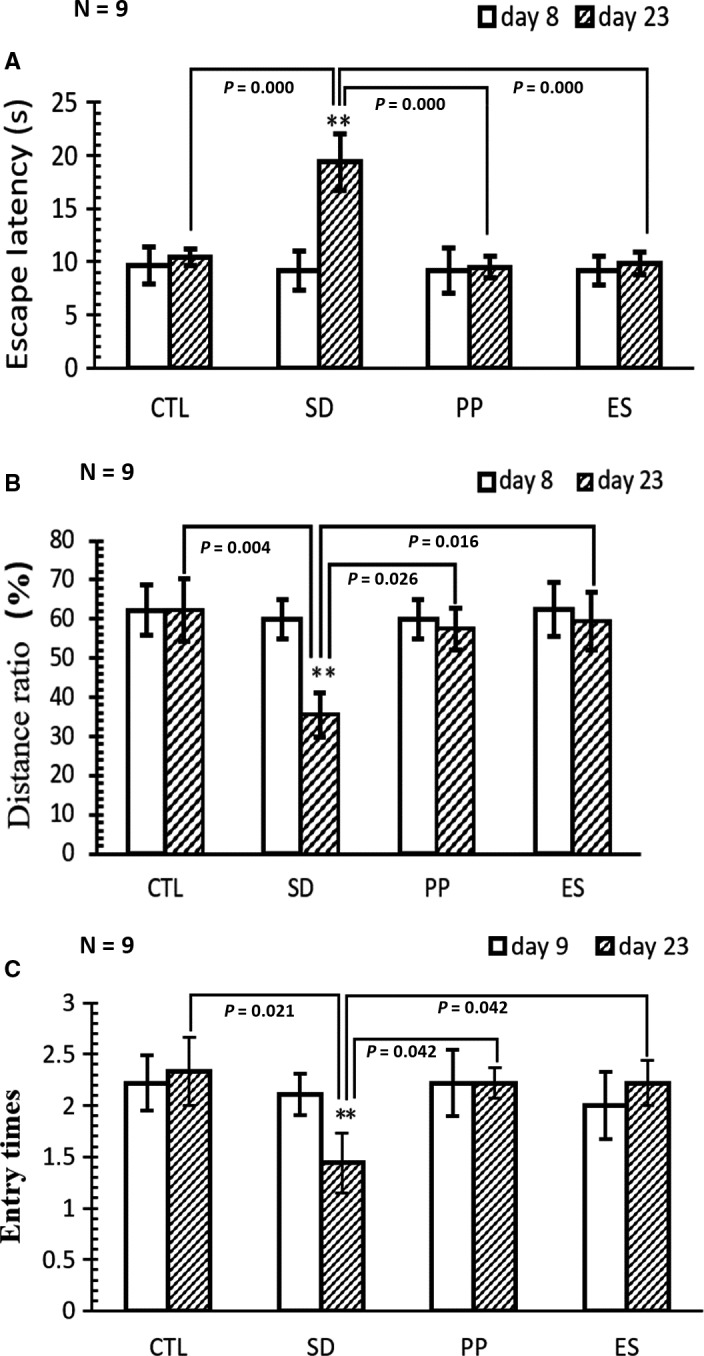
MWM test result comparison before and after modelling (data expressed as mean ± SEM, *p *< .05 represents statistical significant). A, Escape latency is the time (s) in which the rat swims from the starting point to the hidden platform point at the test day (without the hidden platform). The rats in assigned groups (CTR, SD, PP and ES) were tested before (day 8) and after (day 23) modelling (sleep deprivation + treatment). Escape latency in SD was increased significantly from 9.2 ± 1.8 (before modelling) to 19.4 ± 2.6 (after modelling), whereas the escape latencies in the PP and ES groups show no difference before and after modelling. Therefore, sleep deprivation injured the rats’ capability of finding the platform; pearl powder and estazolam reverse the injury. B, Distance ratio: ratio of swimming distance (m) in target quadrant (the hidden platform stood in at the training days) versus total swimming distance in water pool within 60 s is used to reflect the animal memory. The distance ratio in the SD group was significantly decreased from 60.0 ± 5.0 (before SD) to 35.5 ± 5.6 (after SD), whereas the distance ratio of rats in other groups was not significantly changed before and after modelling. Therefore, SD rats were injured in memory, but PP and ES were capable to prevent memory injury. C, Number of entries into the quadrant with withdrawn platform within 60 s as measurement of spatial memory. Before modelling, the rats in different group show the similar entry times: that is, 2.2 ± 0.28 (CTL), 2.1 ± 0.20 (SD), 2.2 ± 0.33 (PP) and 2.0 ± 0.33 (ES). After modelling, the travel times in SD were 1.4 ± 0.27, decreased by 31.8% (*P < .01). However, the travel times in other groups were not changed or slightly increased after modelling. This indicates the rats treated with PP or ES ameliorate spatial memory deficit induced by SD

One‐way ANOVA and Bonferroni post hoc test were used in behavioural tests (Figure 2) and Western blot quantification (Figure 5). Data were expressed as mean ± SEM *P* < .05 is considered as statistically significant difference between groups. To analysis the data of proteomics, paired comparisons of mean number between two groups were performed using *t* test, and the proteins with fold change (ratio) ≥1.2 and those with fold change (ratio) ≤0.83 were considered difference (DEPs) with statistically significant when *P* < .05.

## 3 RESULTS

### 3.1 Behavioural tests

To evaluate the effect of pearl powder on sleeplessness stress, the spatial learning and memory ability were tested using MWM test, which primarily depends on the hippocampus function.^15^, ^16^ Prior to any treatment, all rats were firstly trained for swimming and the measurements were accessed as shown in Figure 2A‐C. All rats exhibited similar behavioural capabilities, that is in a similar latency to reach the position of the hidden platform, and swam approximately at the same ratio of swimming distance in platform quadrant VS total swimming distance in all quadrant within 60 seconds. In modelling period, the SD, PP and ES rats received sleep deprivation stress, whereas those rats also received saline, PP or ES treatment. In the post‐modelling MWM test, compared with CTL, SD rats displayed decreased efficiency searching for hidden platform, while PP and ES rats actively searching for the hidden platform, with efficiency similar to CTL. These results indicate that sleeplessness stress can significantly cause the injury in hippocampus with a shorter latency, decreased the ratio and decreased travel times (%). Furthermore, both PP and ES can significantly improve the spatial learning and memory loss caused by sleep deprivation stress.

### 3.2 Proteomic quality and differential quantitative analysis

**Figure 3:**
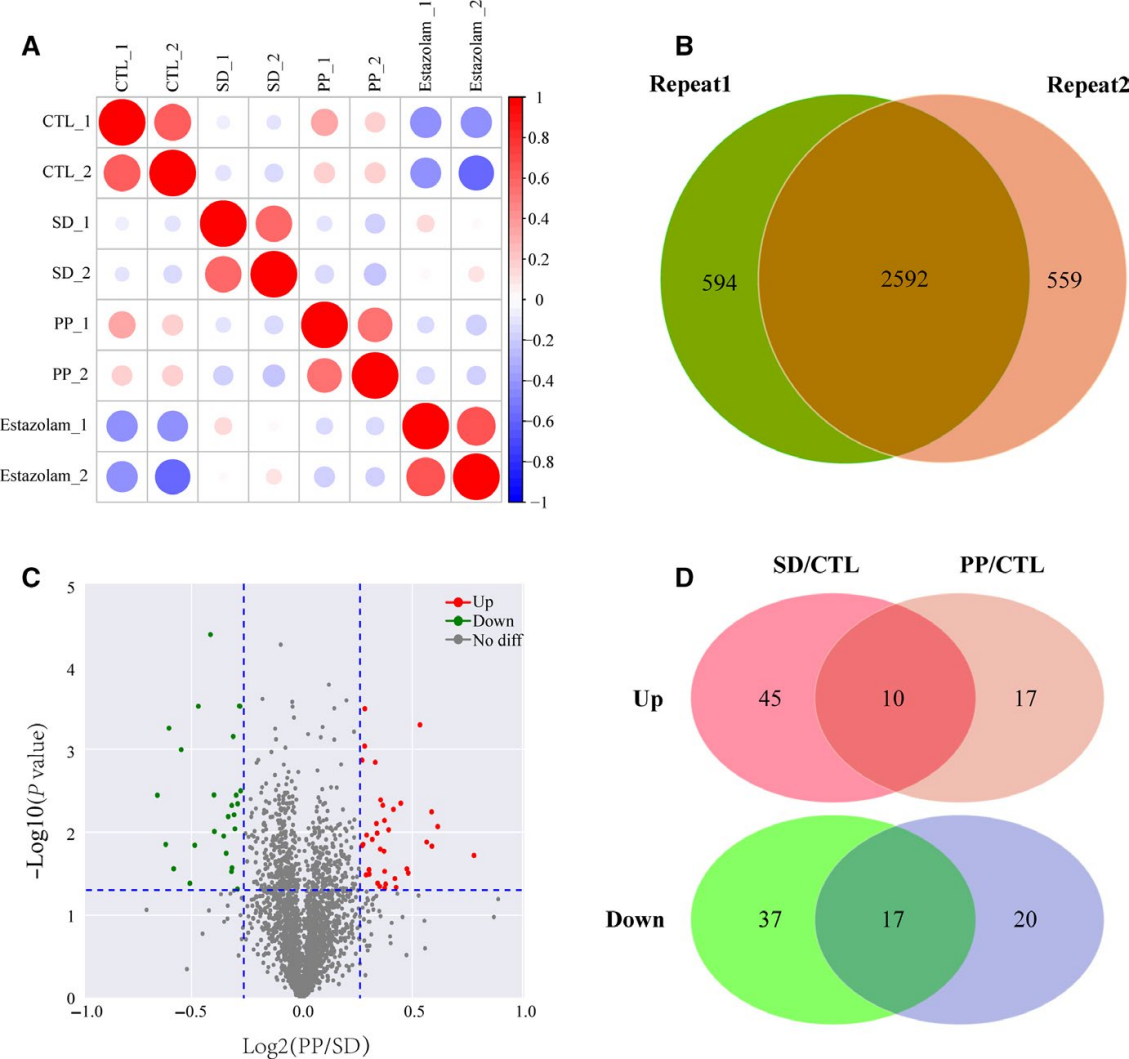
Result overview of differential proteomics based on LC‐MS/MS analysis. A, Repeatability analysis of quantification proteins of two replications. Pearson's correlation coefficient was used for the repeatability analysis. B, Venn diagram of protein identification between each replication. C, DEP screening uses a volcano plots with the threshold of fold change >1.2 or <1/1.2 and *P*‐value <.05. D, Venn diagram showing that 10 up‐regulated and 17 down‐regulated proteins in SD/CTL and PP/CTL overlap

In screening for protein(s) expressed differently from SD vs to other groups in proteomic study, there were total 3745 proteins identified, and 69% (2592 proteins) were quantified with high correlation in biological replicas, and six differential proteins identified from Venn analysis (Figure 3A,B). In total, there were 353 differentially expressed proteins found. Compared with SD, in PP samples, there were 37 proteins were up‐regulated and 27 proteins were down‐regulated (Figure 3C). Compared with the CTL group, 55 proteins in SD group were up‐regulated and 54 proteins were down‐regulated. After pearl treatment, 10 of the 55 up‐regulated proteins were down‐regulated and 14 of the 54 down‐regulated proteins were up‐regulated (Figure 3D). And 22 differential expressing proteins were identified (Table 1). These proteins had different expressing trend after sleep deprivation treatment but rescued towards back to CTL expression direction after pearl was added to these rats.

**Table 1:** The differentially expressed proteins identified in SD vs control group and PP vs SD group

Protein	Description	Gene name	SD‐vs‐CTL	SD‐vs‐CTL	SD‐vs‐CTL	PP‐vs‐SD	PP‐vs‐SD	PP‐vs‐SD
P62716	Serine/threonine‐protein phosphatase 2A catalytic subunit beta isoform	Ppp2cb	1.31	0.002	Up	0.68	0.001	Down
D3ZSP1	Protein LOC100361838	LOC100361838	0.73	0.0038	Down	1.21	0.0145	Up
Q5XIP1	Protein pelota homolog	Pelo	0.76	0.0468	Down	1.27	0.0413	Up
A4GW50	Protein Stk38l	Stk38l	0.78	0.0144	Down	1.29	0.0047	Up
G3V9K0	Cysteinyl‐tRNA synthetase (Predicted), isoform CRA_b	Cars	0.77	0.0041	Down	1.23	0.0284	Up
A0A0H2UHV7	Alanine‐‐tRNA ligase, cytoplasmic	Aars2	0.78	0.0007	Down	1.34	0.0362	Up
D4A4L5	Protein Isca2	Isca2	0.73	0.0114	Down	1.24	0.032	Up
D3ZHV3	Metallothionein	Mt1m	1.28	0.0148	Up	0.82	0.0003	Down
D3ZUI1	Methylthioribulose‐1‐phosphate dehydratase	Apip	1.27	0.0036	Up	0.76	0.0098	Down
M0R660	Glyceraldehyde‐3‐phosphate dehydrogenase	GAPDH	1.21	0.0172	Up	0.82	0.0003	Down
B2RYN1	Fructosamine‐3‐kinase‐related protein	Fn3krp	0.76	0.0018	Down	1.48	0.0131	Up
D3Z8I7	Protein Gstt3	Gstt3	1.22	0.006	Up	0.81	0.0062	Down
F1LMQ3	Protein Psmd8	Psmd8	0.75	0.0291	Down	1.3	0.0476	Up
A0A0G2JZ43	Phosphoinositide phospholipase C	Plcb2	1.26	0.0094	Up	0.82	0.0484	Down
F1LS26	Protein strawberry notch homolog 1	Sbno1	0.25	0.0023	Down	3.5	0.0007	Up
Q6TXI6	LRRGT00013	LOC317456	0.8	0.0029	Down	1.34	0.0464	Up
Q63468	Phosphoribosyl pyrophosphate synthase‐associated protein 1	Prpsap1	0.76	0.0003	Down	1.25	0.0122	Up
D3ZK73	Cullin 4B (Predicted)	Cul4b	0.82	0.0069	Down	1.36	0.0045	Up
F2Z3T7	Isochorismatase domain‐containing protein 1	LOC103694869	0.8	0.0081	Down	1.23	0.0108	Up
D3ZEV0	Protein LOC100912427	LOC100912427	1.23	0.0488	Up	0.19	0.0017	Down
D3ZZN4	Uncharacterized protein	N/A	1.33	0.0297	Up	0.8	0.0299	Down
P05505	Cytochrome c oxidase subunit 3	Mtco3	1.82	0.0073	Up	0.71	0.0143	Down

### 3.3 Functional, pathway and protein domain enrichment analysis

**Figure 4:**
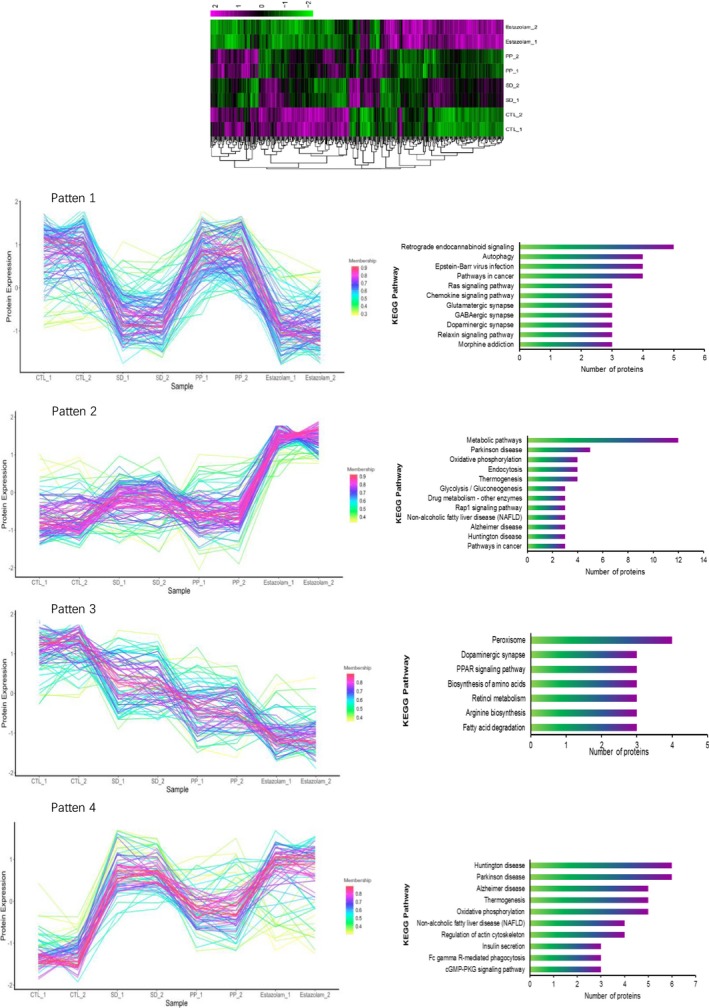
Clustering and KEGG pathway annotation of protein expression pattern based on protein expression level. A, Heatmap of all 353 DEPs resulted from proteomic experiment. B, DEPs were separated to five clusters by protein expression pattern analysis. C, KEGG pathway statistics of proteins in five expression patterns

To obtain a functional overview of differentially expressed protein, gene ontology (GO) annotation analysis was applied. Proteins were functionally classified based on UniProt GO annotation and demonstrated through heat map (Figure 4A). Although as many as 353 differentially expressed proteins were identified, their dynamic changes can be assigned into 5 main patterns (Figure 4B). Furthermore, the differentially expressed proteins in each pattern were applied into pathway analysis through KEGG (Kyoto Encyclopedia of Genes and Genomes) database (Figure 4C). The first pattern represents the populations of protein with decreased expression level in SD groups as compared to CTL, which is rescued in the PP group, but not in the ES group. And the proteins in this pattern were mainly enriched in retrograde endocannabinoid signalling and autophagy pathway. The second pattern shows the populations of proteins with similar expressing level in the SD and PP groups as compared to the CTL group. However, ES treatment caused increased expression suggesting possible side effect caused by ES treatment. The proteins in this pattern were mainly enriched in metabolic pathway, Parkinson disease and oxidative phosphorylation pathway. The third pattern indicates the protein expression level in the SD group is slightly decreased compared with the CTL group. Their expression levels in the PP and ES groups were further decreased suggesting mild side effect caused by PP and ES treatment. The proteins in this pattern were enriched in peroxisome, dopaminergic synapse and PPAR signalling pathway. The fourth pattern demonstrates the groups of protein with increased expressing level in the SD group as compared to CTL. The increase was specifically rescued (partially) to the level similar to CTL in the PP group but not in the ES group. The proteins in this pattern were enriched in Huntington disease pathway, Parkinson disease and Alzheimer disease pathway which all were nerve disease related pathway were most three enriched pathways in this pattern.

### 3.4 Western blot

**Figure 5:**
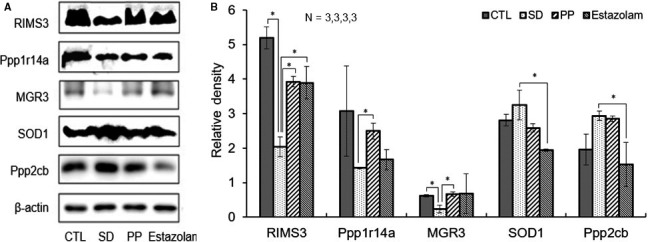
Validation of five differentially expressed proteins in CTL and treatment groups. A, RIMS3, Ppp1r14a, MGR3, SOD1, Ppp2cb and β‐actin bands, respectively, expressed from rat groups of CTL, SD, PP and estazolam. B, The relative density of the validated proteins was normalized with β‐actin as the internal reference. One‐way ANOVA, n = 3, *P < .05

To validate the expression data from proteomic analyses, five proteins related to sleep deprivation were selected for Western blotting assay. Hippocampal protein expression level from four groups of rats (3 rats/group)—Ctr, SD, PP and estazolam—were examined (Figure 5A: representative Western blot result) and quantified (Figure 5B). Of 5 tested proteins, 3 (RIMS3, Ppp1r14a and MGR3) were decreased and 2 (SOD1 and Ppp2cb) were increased in SD as compared to other groups. Western blotting assay was consistent with the results of the mass spectrometry analysis.

### 3.5 Pathway‐protein crosslinking network construction

**Figure 6:**
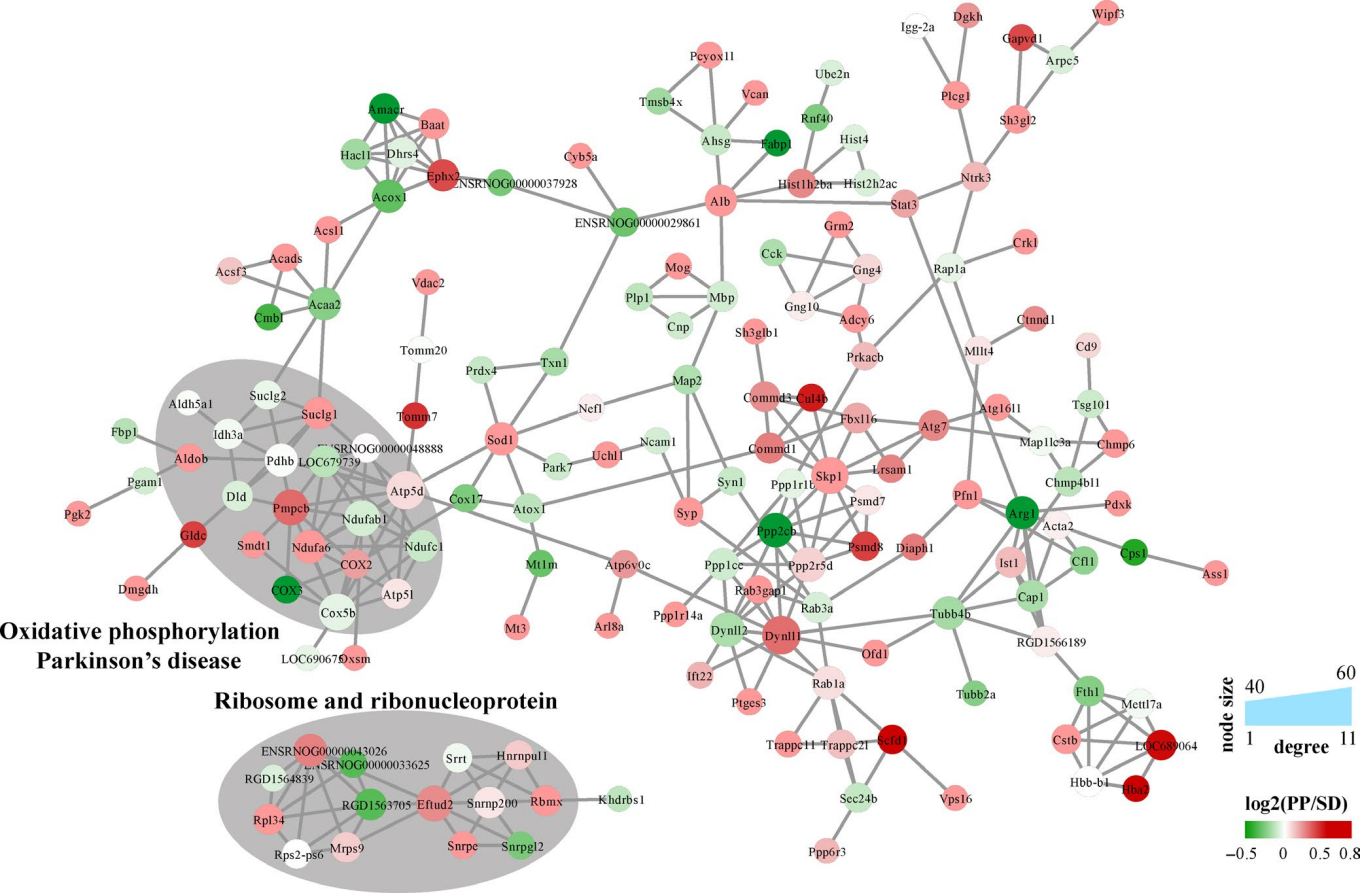
Protein‐protein interaction between DEPs. Proteins' expression ratio of PP/SD was marked with continuous colour mapper. Proteins enriched in oxidative phosphorylation Parkinson's disease and ribosome and ribonucleoprotein were marked in grey cycling

After GO analysis was performed, we would like to explore interaction relationship formed by these differentially expressed proteins. Cytoscape software v2.8.3 (http://www.cytoscape.org) was used to visualize the pathway‐protein crosslinking network (Figure 6). In this network, protein Ppp2cb has most edges which means it has most interaction relationship. Oxidative phosphorylation Parkinson's disease pathway, ribosome and ribonucleoprotein pathway were the two most enriched pathway in this interaction network.

## 4 DISCUSSION

In this study, we used a previously validated rat insomnia model, induced by sleep deprivation, to examine the relationship between SD and learning/memory manifestations. Through test of MWM, we observe that such stress can result in behaviour abnormality which can be rescued by pearl powder—a traditional Chinese medicine that had been used for insomnia. The limitation of this study is that such animal model can only partially recapitulate insomnia in human, which is much more complicated and may be caused by various factors in addition to sleep deprivation (such as gene, drug and various kind of stress).

Impaired hippocampal functions have been implicated in insomnia and cognitive decline. To understand the mechanism how pearl powder affects hippocampus of animals receiving sleep deprivation, we performed a broad screening technology, that is iTRAQ‐based global proteomic. We identified 3745 proteins in stressed rat hippocampus and quantified 2592 proteins for screening. The expression of proteins from SD model with a probability ≥0.95 was obtained via comparisons with control, pearl powder and estazolam samples. Some of the key findings from proteomics were replicated using the Western blot. In Figure 5, proteins changed (down‐regulation of RIMS3, Ppp1r14a and MGR3 and up‐regulation of SOD1 and Ppp2cb) by SD can be effectively reversed by pearl powder in a great extent.

Hierarchical clustering showed that the top 22 temporal proteins and genes that could be divided into four groups exhibited different expression patterns. From the four patterns, we generalized from differentially expressed proteins, the changing trend in the PP group and the ES group was not always the same. This means the pharmacological effect of these two medicines is not the same. Our results indicate that PP shows higher rescue capability, and ES has bigger side effect. Figure 4 shows that pearl powder displays specific rescue effect in pattern 1 and pattern 4. In pattern 1, the pathways most sensitive to PP treatment are retrograde endocannabinoid signalling^17^, ^18^ and autophagy; in pattern 4, the most sensitive pathways to PP treatment are the proteins enriched relative to Huntington disease and Parkinson disease (pattern 4).

Our experimental results have human correlates with studies that support the application of pearl powder to stressful behaviour disorder.^19^ There is substantial literature correlating depression symptoms and psychosocial dysfunction in patients with insomnia.^20^, ^21^, ^22^, ^23^ More direct evidence also comes from a randomized placebo‐controlled trial showing that pearl powder may promote the development of antioxidant capacity and cognitive hypervigilance.^24^ It has also been suggested that humans who experience an adverse life event have a hyper‐responsive HPA and that HPA reactivity may in turn modulate memory and learning impairment.^25^

It is known that chronic insomnia patients show abnormal substructure,^26^ which may be caused by decreased neurogenesis.^27^ Endocannabinoid signalling (ECS) is altered in preclinical and clinical models of depression, with one of the common symptoms as insomnia. Several studies indicating that hippocampal progenitor proliferation and neurogenesis require intact ECS.^28^, ^29^ Therefore, ECS pathway deficit is one of the underline mechanisms for abnormal hippocampal structure and activity in insomnia patients, which can be rescued by pearl treatment.

Further studies will be required to understand the mechanisms regarding the regulation of hippocampus such as impaired feedback signalling from epigenetic changes regulating RIMS3, Ppp1r14a, MGR3, SOD1 and Ppp2cb or other forms of expression. Such as the impact of pearl treatment on endocannabinoid signalling pathway in hippocampus of SD rats. What initiates this cascade of events during stress is also unknown. Insomnia may alter the HPA axis through activation of cytokines, which is another biological targeted that may be rescued by pearl powder.

## 5 CONCLUSIONS

In conclusion, our results demonstrate, for the first time, that sleep deprivation causes hippocampus injury through several pathways including endocannabinoid signalling, autophagy, Huntington disease, oxidative phosphorylation Parkinson disease and ribosome and proteasome, which can be corrected (at least partially) by pear powder. Clinically, the cause of insomnia is very complicated, and pearl powder had been used in combination with other Chinese medicine as treatments for insomnia, with unclear functionality. Interestingly, the identified pathways had been implicated in the pathology of anxiety and depression. Therefore, we speculate that pearl powder by itself may be useful for anxiety‐ or depression‐induced insomnia. Further studies can be focused on dissecting effect of pearl powder on each biological pathway, in the context of insomnia and cognitive decline.

## CONFLICT OF INTEREST

All authors declare no conflict of interests.

## AUTHOR CONTRIBUTIONS

Jiang Lin designed the study. Meng Xia, Delun Huang and Yuangming Tong performed experiments. Meng Xia analysed the data. Delun Huang and Yuangming Tong carried out critical revision of the manuscript for important intellectual content.

## Data Availability

The data used to support the findings of this study are available from the corresponding author upon request.
